# Variation in the Untranslated Genome and Susceptibility to Infections

**DOI:** 10.3389/fimmu.2018.02046

**Published:** 2018-09-07

**Authors:** Veron Ramsuran, Rodger Ewy, Hoang Nguyen, Smita Kulkarni

**Affiliations:** ^1^Centre for the AIDS Programme of Research in South Africa, KwaZulu-Natal Research Innovation and Sequencing Platform, School of Laboratory Medicine and Medical Sciences, Nelson R. Mandela School of Medicine, College of Health Sciences, University of KwaZulu-Natal, Durban, South Africa; ^2^Genetics Department, Texas Biomedical Research Institute, San Antonio, TX, United States

**Keywords:** methylation, promoter, microRNA, lncRNA, polymorphism

## Abstract

The clinical outcomes of infections are highly variable among individuals and are determined by complex host-pathogen interactions. Genome-wide association studies (GWAS) are powerful tools to unravel common genetic variations that are associated with disease risk and clinical outcomes. However, GWAS has only rarely revealed information on the exact genetic elements and their effects underlying an association because the majority of the hits are within non-coding regions. Some of the variants or the linked polymorphisms are now being discovered to have functional significance, such as regulatory elements in the promoter and enhancer regions or the microRNA binding sites in the 3′untranslated region of the protein-coding genes, which influence transcription, RNA stability, and translation of the protein-coding genes. However, only 3% of the entire transcriptome is protein-coding, signifying that non-coding RNAs represent most of the transcripts. Thus, a large portion of previously identified intergenic GWAS single nucleotide polymorphisms (SNPs) is in the non-coding RNAs. The non-coding RNAs form a large-scale regulatory network across the transcriptome, greatly expanding the complexity of gene regulation. Accumulating evidence also suggests that the “non-coding” genome regions actively regulate the highly dynamic three dimensional (3D) chromatin structures, which are critical for genome function. Epigenetic modulation like DNA methylation and histone modifications further affect chromatin accessibility and gene expression adding another layer of complexity to the functional interpretation of genetic variation associated with disease outcomes. We provide an overview of the current information on the influence of variation in these “untranslated” regions of the human genome on infectious diseases. The focus of this review is infectious disease-associated polymorphisms and gene regulatory mechanisms of pathophysiological relevance.

## Introduction

The influence of host genetic polymorphisms on inter-individual variation in disease outcomes has been investigated through candidate gene sequencing and the large scale Genome-wide association studies (GWAS). However, these studies have rarely revealed information on the exact genetic elements underlying an association because majority of the hits are in intergenic regions, some of which are now being discovered to have functional significance. This lack of functional information limits their use as biomarkers or potential targets for therapy.

Despite the high resolution and specialized genotypic platforms and the wealth of data, which allows genotype imputations (1), causal genetic variations have remained elusive. Surveys of several published GWAS indicate a multitude of disease-associated polymorphisms in enriched in non-coding regions (2), regulatory elements (3) and expression quantitative trait loci (eQTL) (3, 4), suggesting that their associations with diseases are due to their involvement in regulation of gene expression. Only about 10% of the disease associated single nucleotide polymorphisms (SNPs) in GWAS affect protein-coding sequences (2). The majority of non-coding SNPs are in the deoxyribonuclease I (DNase I) hypersensitive sites (2). These SNPs may affect binding of transcription factors to the promoter and enhancer regions and transcription of the downstream genes. It is complicated to delineate functions of enhancer region polymorphisms as they can be defined mostly by the epigenomic profile like methylation, chromatin accessibility, histone modifications and expression of enhancer RNAs (eRNAs)(2, 5–7). 3D chromatin structure has only begun to be studied since the turn of the millennium beginning with the development of the chromosome conformation capture (8). Growing literature indicates that disease associated SNPs in the non-coding genome contribute to gene regulation through 3D interactions (9, 10). A small percent of SNPs in the 3′untranslated region (3′UTR) influence post-transcriptional mRNA decay or translation by altering the microRNA (miRNA) binding sites.

Recently, another class of non-coding RNAs, termed long non-coding RNAs (lncRNAs) have been discovered. The lncRNAs regulate a number of cellular and developmental processes (11–13). The majority of the transcriptome is made up of these non-coding RNAs. Thus, it is predicted that a sizable portion of previously identified intergenic GWAS SNPs could be marking variation in function or expression of non-coding RNAs (14, 15).

Here we will review the influence of variation in the non-coding genome on transcriptional or post-transcriptional regulation of gene expression and infectious disease outcomes.

## Promoter regulatory elements

Polymorphisms in the DNA regulatory regions modulate the epigenetic alterations and transcription factor binding resulting in variations in gene expression and immune responses. Cytokines, chemokines and their receptors are the key regulators of immune response and inflammation. Promoter polymorphisms in these genes are associated with several infectious diseases. Interleukin 10 (*IL-10)* promoter polymorphism is associated with increased mortality in severe sepsis (16), susceptibility to chronic hepatitis C virus (HCV) infection, resistance to antiviral therapy (17), and predisposition to Epstein Barr virus (EBV) infection (18). *IL-8* promoter polymorphisms is associated with IL-8 release and incidence of virus bronchiolitis (19). Genomic variations in the promoters of cytokines and other innate immune genes have been linked to susceptibility to *Mycobacterium tuberculosis* (*M.tb*) infection (20). Promoter variations in the chemokine (21) and chemokine receptor genes are known to influence the course of HIV infection (22–24). Polymorphism in a TF binding site of *HLA-C* promoter associated with HIV control (25).

Some of the gene regulatory polymorphisms alter the DNA methylation pattern. A methyl group is added to the nucleotide cytosine, which is followed by a guanine to form a CpG dinucleotide (26). Short stretches of DNA with frequent CpG dinucleotides termed CpG islands are mainly located near the promoters of genes. Variation in the promoter methylation of *CCR5* (27) and human leukocyte antigen (*HLA-A)* genes have been shown to significantly impact outcomes of human immunodeficiency virus (HIV) infection (28).

## Splicing

Precise splicing of mRNAs is critical for its translation and functioning of the resulting protein. Alternative splicing is often employed by the cells to generate transcript diversity (29, 30). Splicing is orchestrated by the complex interaction between spliceosomes and intronic splicing signals. Spliceosomes are complexes of small ribonucleoproteins (snRNPs), which interact with intronic splicing signals like donor and acceptor sites, polypyrimidine tract, branch points like enhancers and silencers of splicing. Sequence variation in these splicing signals affect mRNA processing. A wide range (15–60%) of the human disease related polymorphisms are predicted to alter splicing (31). A SNP in the acceptor site of an antiviral enzyme *OAS1* associates with the level of OAS1 activity and susceptibility to viral infections (32). Intron region polymorphism in *ULK1* associate with decreased expression of the gene, compromised immune responses and associate with increase *M.tb* replication in the latently infected patients leading to the development of pulmonary TB (33). The SNPs in the splice sites of *PLCXD3* showed significant association with prion mediated sporadic Creutzfeldt-Jakob's Disease (34).

## MicroRNA

MicroRNAs are small, 22 nucleotide RNAs associated with RNA-induced silencing complexes (RISC) and target specific mRNAs for degradation or inhibition of translation. The genomic variation in miRNA or the miRNA binding site in the target genes have been implicated in the differential susceptibility and clinical manifestations of infectious disease. The SNPs in host miRNA loci have been associated with susceptibility to leprosy, clearance of hepatitis B virus (HBV), human cytomegalovirus (hCMV) infection (35–39), the prion mediated spontaneous Creutzfeldt-Jakob's Disease and fatal familial insomnia (40).

A mutation in the miRNA binding site can disrupt binding to miRNA to its target thus allowing the target to be expressed at higher levels. MicroRNA binding site polymorphisms have been implicated in susceptibility or prognosis of infection. The functional effect of some of these associations have been validated. High levels of *HLA-C* mRNA and cell surface expression associate with control HIV viremia and slower progression to acquired immunodeficiency syndrome (AIDS) (41, 42). The allele specific expression variation of *HLA-C* is partly explained by a polymorphic miR-148a binding site encoded in the 3′ UTR of *HLA-C*. The alleles with disrupted miRNA binding site escape regulation by miR-148a, are expressed at higher levels and associate with lower HIV viral loads (43). A functional SNP, within the 3′ UTR of *IFNL3* is in a binding site of HCV-induced cellular miRNAs. The allele, which allows escape of miRNA mediated downregulation associates with an increase in *IFNL3* miRNA expression and showed significant association with natural and therapy-induced HCV clearance (44).

Host miRNAs target the HIV transcripts and inhibit translation resulting in silencing of HIV gene expression in resting CD4+ T cells (45), whereas HCV requires host miR-122 for replication (46). Pathogens deregulate the host miRNA expression to their advantage, such as in Zika virus in astrocytes (47), *Mycobacterium leprae* as well as *M. tb* infection (48, 49); or directly target the host transcriptome with a miRNA encoded in their own genome, such as in Rotavirus (50).

## Long non-coding RNAs

The long non-coding RNAs are >200 bp transcripts without protein-coding potential. LncRNAs have been shown to enhance or repress the transcription of protein-coding genes, including immune-related genes (51–53), implicating a role for lncRNAs in disease outcomes through gene regulation. The majority of lncRNAs are found in low abundance and localized in the nucleus (54), where they participate in transcriptional regulation through diverse mechanisms (55, 56). However, some localize to the cytoplasm, where they function as competitive endogenous RNAs that can act as “sponges” for miRNAs (57). Thus, the lncRNAs form a large-scale regulatory network across the transcriptome (58). LncRNA gene variations are associated with disease outcomes, and molecular mechanisms have been delineated for a few in cancer (59) and autoimmune disease (60). A SNP in a lncRNA expressed in neutrophils was associated with doubled risk of *Pneumococcal bacteremia* in Kenyan children (46). Polymorphisms in lncRNAs are associated with tuberculosis susceptibility (61). A variant in the lncRNA gene has been shown to confer susceptibility to HBV-related carcinoma in Chinese population (62). Several viruses encode lncRNAs in their genome (63) and modulate host responses. A few known examples are arthropod-borne flaviviruses (64), Kaposi's sarcoma-associated herpesvirus (65) and HIV (66–68). Pathogens like influenza A (69), HCV (70), adenovirus (71), herpes simplex virus [HSV-1] (72), HIV (13, 73–75) and *M.tb* (76–80) alter expression of the host lncRNAs upon infection. An interferon-independent host lncRNA promotes viral replication by modulating cellular metabolism (81). The expression and functional variations within the lncRNA genes and their role in regulating immune response and inflammation is an area of intense research and likely to discover novel pathways of host-pathogen interactions.

## Multidimensional regulation and complex gene interactions

**Figure 1:**
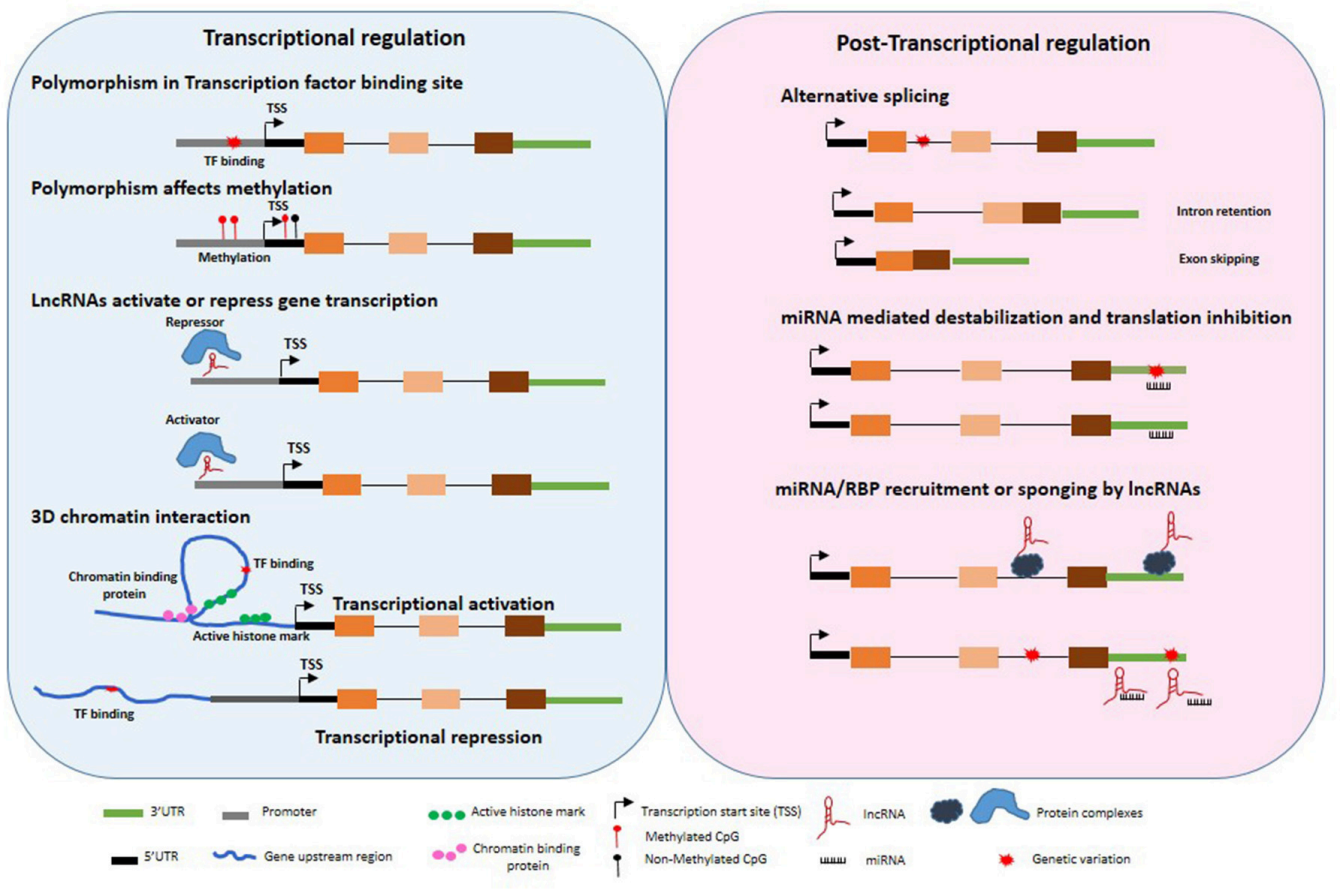
Untranslated gene variations influence regulation of gene expression. Disease associated polymorphisms may alter methylation, transcription factor binding in the gene promoter regions, recruitment of repressor or activators, 3 dimensional chromatin structure, splicing, miRNA binding to 3′UTR, transcriptional and post-transcriptional regulation of target genes through variation in lncRNA expression and function.

The variation in gene expression levels are determined by several transcriptional, post-transcriptional and post-translational processes (Figure 1). A single variant or haplotype may not be the sole contributor to disease associations. This is exemplified by a few genetic associations for which molecular mechanisms have been delineated.

GWAS implicated multiple linked variations at the interferon lambda (*IFNL*) in HCV clearance (82, 83). A 3′UTR variant in *IFNL3* disrupting miRNA binding site and increasing stability of *IFNL3* mRNA was found to be protective (44). A strongly linked dinucleotide variant, which introduces a premature stop codon in another interferon gene, *IFNL4* associated with HCV clearance (84). Thus linked genotypes conferring high expression of IFNλ3 and truncated, non-functional IFNλ4 associated with HCV clearance. This paradoxical association of expression of the full length antiviral protein IFNλ4 with HCV persistence remained unexplained. Recently, it was found that the full length IFNλ4 isoform is poorly expressed due to aberrant splicing resulting in intron retention and a weak polyadenylation signal (85). The mRNA instability genotype in the *IFNL3* 3′UTR is in strong linkage disequilibrium (LD) with the full length expression genotype of *IFNL4* suggesting that the persistence of HCV is likely attributed to the impaired expression and anti-viral function of IFNλ3.

The *HLA* genes located on human chromosome 6 are extremely polymorphic, exhibit strong LD and allele-specific expression patterns. The nucleotide diversity observed within the *MHC* region, particularly within the regulatory region upstream of the initiation codon of certain class I genes, has been observed about 20-fold higher than the genome average (86). Variations within the *HLA-C* 3′UTR (43) and promoter (25) showed a strong influence on *HLA-C* mRNA and protein expression, which associates with HIV control. Both the causal variations in the 3′UTR and promoter only partially account for the allele specific gene expression variation, indicating that yet unknown variation either within the *HLA-C* gene region or elsewhere in the genome is responsible for a large part of HLA-C expression.

## Interplay between genomic variations and epigenomic modulations

Specific epigenetic changes could result in switching on or off certain genes, and determines which proteins are transcribed. In some cases, several factors have been shown to contribute to causal phenotype in addition to the genomic variation. A recent study investigated role of a SNP in 5′UTR of the gene encoding an antiviral protein, Interferon-induced transmembrane protein 3 (IFITM3), which inhibits entry of influenza virus into the host cell. The risk allele was found to disrupt a CpG site, decrease binding of a transcription factor, interferon regulatory factor (IRF3) and increase binding of CCCTC-binding factor (CTCF) leading to lower expression of IFITM3 expression, altered methylation and also affected the expression of *IFITM3* neighboring genes important in anti-viral response. CTCF is a versatile protein important in maintaining topologically associating domains of chromatin and contributing to 3D chromatin structure. CTCF has also been shown to function as an “insulator” blocking chromatin positioning and inhibiting interaction of enhancer with promoters. Increased CTCF binding associated with the IFITM3 risk allele and disruption of neighboring gene expression patterns indicative of CTCF boundary activity.

**Figure 2:**
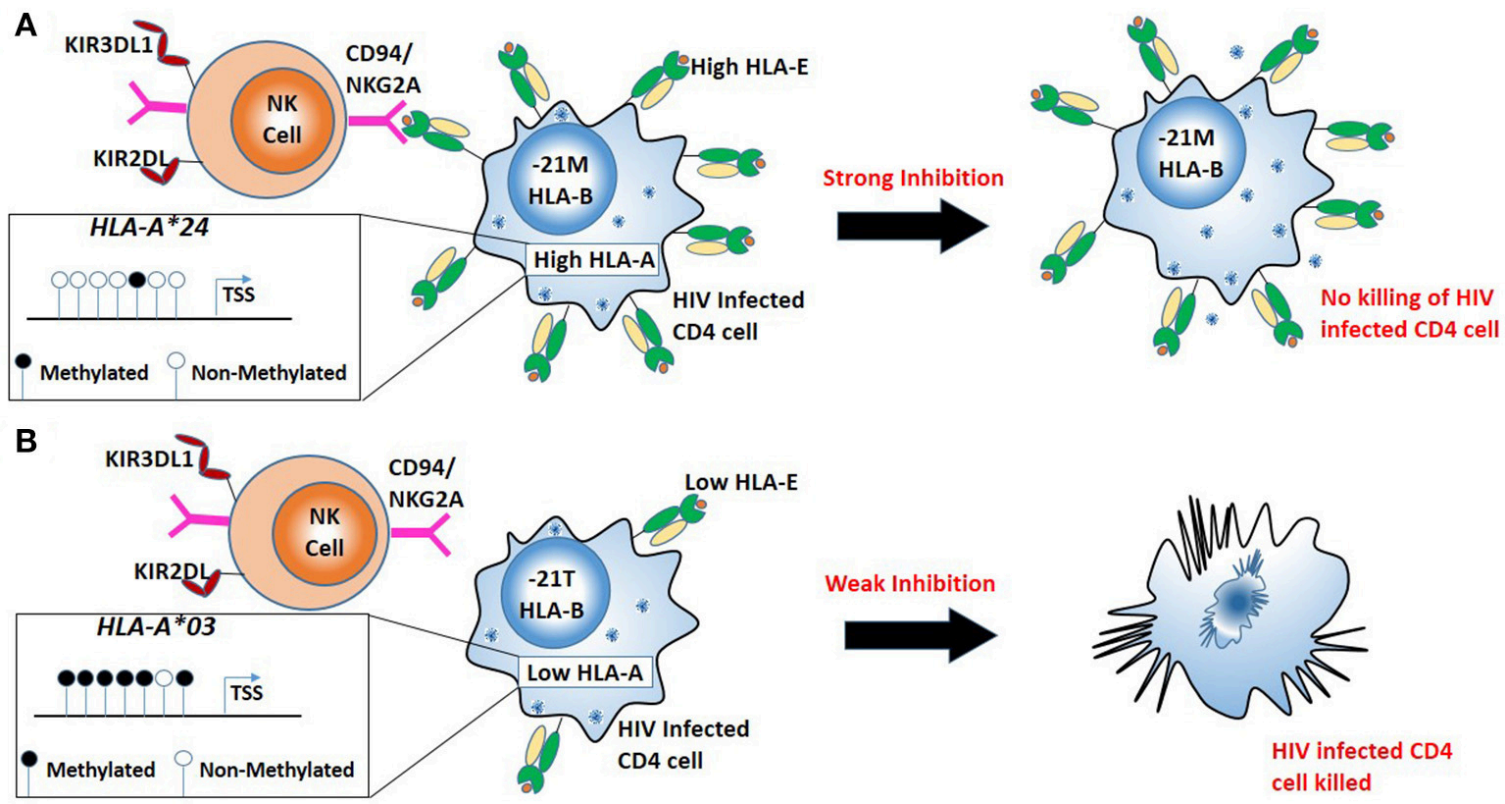
Combination of high HLA-A allele expression and HLA-B alleles mediating in robust “licensing” of NK cells result in strong NK cell inhibition and result in poor HIV control. **(A)** Less promoter methylation results in a high expression HLA-A (e.g., HLA-A*24). High HLA-A expression with the−21M genotype at *HLA-B* leads to increased cell surface expression of HLA-E, robust NK cell licensing through CD94/NKG2A increased inhibition of NK cells. Strong inhibition of NK cells leads to escape of HIV infected cells from NK mediated killing. **(B)** High promoter methylation results in low expression of HLA-A (e.g., HLA-A*03). Low expression *HLA-A* allele with−21T genotype at *HLA-B* results in lower HLA-E on the surface resulting in little CD94/NKG2A licensing, NK cells mediated killing HIV infected cells.

DNA methylation and histone modification are just two of the epigenetic mechanisms widely described (87). Examining certain genes, we observe the balancing act between genomics and epigenomics within infectious diseases. One such example is the *HLA-A* gene, where specific *HLA-A* alleles are observed to possess individual HIV allelic effects, based on the peptide presented (88). However, more recently it has been shown *HLA-A* alleles possess distinct allele-specific promoter methylation and mRNA expression levels (89). Furthermore, examining nearly 10,000 HIV patients across diverse ethnic backgrounds, Ramsuran *et al* showed that *HLA-A* mRNA expression levels play an important role in HIV viral control (28). The patients with high expression of *HLA-A* showed impaired HIV control. Unlike the protective effect of high HLA-C expression on HIV viral control and contrary to conventional understanding, elevated levels of *HLA-A* do not protect against HIV but rather lead to accelerated disease progression by increasing cell surface expression of a natural killer (NK) ligand, HLA-E and stronger inhibition of NK cells (Figure 2). HLA-E serves as a ligand for the strong inhibitory NK receptor CD94/NKG2A. A signal peptide derived from the leader sequence of *HLA-A, -B* and *-C* molecules stabilizes HLA-E expression on the cell surface (90). The signal peptide has a variation at position 2 (residue−21) resulting in either a methionine (-21M) or threonine (-21T). A methionine (-21M) stabilizes and increases HLA-E expression on the cell surface, while the presence of−21T results in lower HLA-E expression. High expression of HLA-A provides elevated levels of HLA-A derived signal peptide increasing HLA-E expression resulting in strong NK inhibition and impaired killing of HIV infected cells. Presence of HLA-B alleles with−21M results in strong “licensing” of NK cells and further exacerbates the inhibitory effects of high HLA-A expression on NK cell activity and results in poor HIV control. Thus, variable HLA-A expression levels in combination with either−21M or −21T HLA-B alleles lead to diverse HIV outcomes (28).

Variability within the *HLA-A* peptide binding region (exons 2 and 3) of the genomic sequence allows specific alleles to slow or accelerate HIV disease progression. However, epigenetic mechanisms alter the expression levels of these alleles, which also causes delayed or rapid HIV progression. This balance between genetic and epigenetic events makes this region one of the most complex multifactorial sites associating with a range of diseases.

## 3D chromatin structure

Over half of the significant associations in the published GWAS were reported in genome expanses lacking annotated genes in the large regions (>500 kb) flanking associated SNP. Such regions, termed “gene deserts” have been implicated in the regulation of genome function through their influence on intricate 3D genome structure. The 3D architecture of the genome facilitates spatial juxtaposition of and interaction between distant loci, which contributes to gene regulation. A SNP on chromosome 8q24 in a gene desert of 1.2Mb has been shown to affect long range (>300Kb) interactions of a cancer gene, Myc (9, 10). SNPs associated with type 2 diabetes have been implicated in 3D genome interactions leading to impaired interferon signal responses (10). Although 3D chromatin structure changes after infection have been reported in a few cases (91–95), studies on 3D genome structure are underrepresented in the field of infectious diseases.

## Perspective and future directions

Gene association studies and GWAS have identified several genetic variants, which influence outcomes of complex human disease including infections. However, the majority of the disease-associated variants map to the non-coding regions of the genome. Discovery and functional validation of causal variants and the specific regulatory mechanisms are needed to advance understanding of the molecular pathways and their possible manipulation by therapy. Current literature reveals a number of efforts in determining the causal SNPs from the variants identified in the GWAS and linked SNPs. Disease-associated SNPs are further investigated to determine if they are marking coding-region variants through strong LD or are likely to alter promoter and enhancer region, splicing, miRNA binding, or lncRNA expression and function. The possible candidates are examined in a variety of *in silico* and molecular and biochemical analyses, including prediction of transcription factor or miRNA binding sites, chromatin or RNA immunoprecipitations, nucleic acid sequencing and mass-spectrometry to identify DNA, RNA or protein factors interacting with the genomic variation or the resulting RNA and protein products. Loss or gain of function studies are carried out to determine functional consequences of the RNA or protein target. Distinct regions in the genome (promoter, 3′UTR, intron, miRNA, lncRNA) require diverse molecular and biochemical techniques. Although these focused, gene-specific studies discover novel molecular mechanisms of gene regulation, a fresh approach is needed to detangle multilayered gene regulatory pathways and complex gene interactions, which are often cell-type and context-specific (infected vs. uninfected).

Interpretation of the functional consequences of a disease associated gene variation can be complex, especially in case of the intergenic SNPs as it may affect none or multiple genes and pathways through the expression of non-coding RNAs and chromatin organization. CRISPR/Cas9 mediated editing of the SNP or the putative target region is being increasingly used to address the ambiguity. Cell line based models are more permissive for such manipulations. However, these models may not represent the complex environment of cells and tissues. Organoid models are being developed to recapitulate the tissue environment and interaction. Multiple genetic variants co-operatively regulate a phenotype (96) Editing of a single gene will not discern this combinatorial effect. Further evolution of gene editing techniques to simultaneously target multiple regulatory regions is needed of the gene editing techniques to simultaneously target multiple regulatory regions. Gene editing techniques are even harder to implement in case of long non-coding RNAs. Manipulation of gene expression through short interfering RNAs (siRNA, shRNAs) and nucleases are limited by off-target effects. Alternatively approaches of nuclease deficient CRISPR/Cas9 (dCas9) mediated inhibition or activation of gene expression are being increasingly applied specially in the field of non-coding RNAs. Epigenetic modulations are hard to recapitulate as the causal and the target regions could be spread over a large genomic distance. The engineered systems utilizing dCas9 collectively called “epigenetic toggle switches” are being used to mimic deletion of enhances or recruitment of epigenetic repressors (97). Full implications of variations that influence 3D chromatin structure are yet to be realized in part due to difficulties in precisely capturing and mapping interactions between distinct regions of chromatin. However, the field has exploded with multiple complementary techniques like Hi-C, ChIA-PET, 3D-FISH, which help capture both the short and the long-range interactions (98). Integrated investigations of disease-associated genetic variations, context-specific gene expression, spatial organization of chromatin and epigenetic alterations in primary human cells, genomic and transcriptomic analysis at tissue and single cell level are needed to unravel the role of untranslated regions in susceptibility to infections.

## Author contributions

VR and SK conceptualized the framework and all authors contributed to writing, reviewing and editing of the review.

### Conflict of interest statement

The authors declare that the research was conducted in the absence of any commercial or financial relationships that could be construed as a potential conflict of interest.
